# Impact of Helminth Diagnostic Test Performance on Estimation of Risk Factors and Outcomes in HIV-Positive Adults

**DOI:** 10.1371/journal.pone.0081915

**Published:** 2013-12-04

**Authors:** Michael B. Arndt, Grace John-Stewart, Barbra A. Richardson, Benson Singa, Lisette van Lieshout, Jaco J. Verweij, Laura R. Sangaré, Loice W. Mbogo, Jacqueline M. Naulikha, Judd L. Walson

**Affiliations:** 1 Department of Epidemiology, University of Washington, Seattle, Washington, United States of America; 2 Department of Global Health, University of Washington, Seattle, Washington, United States of America; 3 Department of Medicine, University of Washington, Seattle, Washington, United States of America; 4 Department of Pediatrics, University of Washington, Seattle, Washington, United States of America; 5 Department of Allergy and Infectious Disease, University of Washington, Seattle, Washington, United States of America; 6 Department of Biostatistics, University of Washington, Seattle, Washington, United States of America; 7 Centre for Clinical Research, Kenya Medical Research Institute, Nairobi, Kenya; 8 Centre for Reproductive Health, Kenya Medical Research Institute, Nairobi, Kenya; 9 Department of Parasitology, Leiden University Medical Centre, Leiden, The Netherlands; Federal University of São Paulo, Brazil

## Abstract

**Background:**

Traditional methods using microscopy for the detection of helminth infections have limited sensitivity. Polymerase chain reaction (PCR) assays enhance detection of helminths, particularly low burden infections. However, differences in test performance may modify the ability to detect associations between helminth infection, risk factors, and sequelae. We compared these associations using microscopy and PCR.

**Methods:**

This cross-sectional study was nested within a randomized clinical trial conducted at 3 sites in Kenya. We performed microscopy and real-time multiplex PCR for the stool detection and quantification of *Ascaris lumbricoides*, *Necator americanus, Ancylostoma duodenale, Strongyloides stercoralis*, and *Schistosoma* species. We utilized regression to evaluate associations between potential risk factors or outcomes and infection as detected by either method.

**Results:**

Of 153 HIV-positive adults surveyed, 55(36.0%) and 20(13.1%) were positive for one or more helminth species by PCR and microscopy, respectively (p<0.001). PCR-detected infections were associated with farming (Prevalence Ratio 1.57, 95% CI: 1.02, 2.40), communal water source (PR 3.80, 95% CI: 1.01, 14.27), and no primary education (PR 1.54, 95% CI: 1.14, 2.33), whereas microscopy-detected infections were not associated with any risk factors under investigation. Microscopy-detected infections were associated with significantly lower hematocrit and hemoglobin (means of -3.56% and -0.77 g/dl) and a 48% higher risk of anemia (PR 1.48, 95% CI: 1.17, 1.88) compared to uninfected. Such associations were absent for PCR-detected infections unless infection intensity was considered, Infections diagnosed with either method were associated with increased risk of eosinophilia (PCR PR 2.42, 95% CI: 1.02, 5.76; microscopy PR 2.92, 95% CI: 1.29, 6.60).

**Conclusion:**

Newer diagnostic methods, including PCR, improve the detection of helminth infections. This heightened sensitivity may improve the identification of risk factors for infection while reducing ability to discriminate infections associated with adverse clinical outcomes. Quantitative assays can be used to differentiate infection loads and discriminate infections associated with sequelae.

## Introduction

The burden of soil-transmitted helminth infections and schistosomiasis is considerable; there are over a billion infections globally, with more than half of these infections occurring in Sub-Saharan Africa[1]. Helminth infections, including schistosomiasis, are a significant source of morbidity; contributing to iron deficiency/anemia[2], growth and cognitive deficiencies,[3], impaired Vitamin A absorption[4], and reduced economic productivity[5,6].

Helminth infections have traditionally been detected using stool microscopy techniques, which have high specificity but limited sensitivity[7-10], especially in populations where infection intensity (based on egg excretion) is low. Newer assays, including polymerase chain reaction (PCR), have higher sensitivity and high specificity and enhance detection of helminth infections[11-13].. As these newer diagnostic assays become more available and are more widely used, it is important to determine the impact that such testing will have on our understanding of the risk factors and consequences of helminth infection.

Studies using microscopy to evaluate risk factors associated with helminth infections have found associations with age[14-20], gender[16,17], education[15,19,21,22], farming occupation[22,23], rural habitation[22,24,25] and poor hygiene practices[16]. In addition, microscopy-identified helminth infections have been associated with anemia[2,26-28], micronutrient deficiency[4], reduced physical fitness and worker productivity[5,6]. Real-time PCR detected *A. duodenale* infections have also been associated with iron deficiency and severe anemia in pre-school children[29]. 

In the present study we investigated differences in factors associated with helminth infections detected by microscopy versus PCR in a cohort of HIV-positive Kenyan adults. We hypothesized that identification of risk factors and clinical outcomes associated with helminth infection would be influenced by diagnostic method. As HIV-infection may alter excretion of parasite eggs in stool due to immunodysregulation[30], we also assessed the impact of immune status (as measured by CD4 count level) on the ability of these assays to detect helminth infection.

## Methods

### Ethics statement

The UW Human Subjects Review Committee and the KEMRI Ethical Review Committee approved the study protocol, including the use of oral consent, and the trial was registered (ClinicalTrials.gov, number NCT00507221). Participants provided written consent in their preferred language (Kiswahili, Kisii, Luo, Giriama, or English) or if illiterate, gave oral consent in the presence of a witness and confirmed by thumbprint.

### Study design

The study utilized a cross-sectional design nested within a 2-year randomized clinical trial, the Helminth Eradication to delay ART Trial (HEAT) study. HEAT compared an anthelmintic regimen consisting of single dose albendazole (400 mg) every three months and praziquantel (25 mg/kg) given annually to standard care among ART (antiretroviral therapy) naïve, HIV infected adults in Kenya. The methods and results of this clinical trial have been previously published[31].

### Study subjects

HIV-positive individuals were recruited from clinics at three sites in Kenya (Kisii Provincial Hospital, Kisumu District Hospital, and Kilifi District Hospital) between February 2008 and June 2010. Individuals were included if they were aged 18 years or older, were HIV seropositive, and did not meet WHO criteria for ART initiation (on the basis of disease stage and CD4 cell count). Exclusion criteria included pregnancy at enrolment, anthelminthic use in the previous 6 months, or prior ART use (except for the prevention of mother-to-child transmission). 

### Data collection

Socioeconomic and demographic information was collected from individuals at enrollment. At enrollment and every 6 month study visit, all individuals in the RCT had full blood counts with differential assessed at study site, CD4 counts assessed by FACSCalibur at the KEMRI/UW Flow Laboratory at the Centre for Clinical Research in Nairobi, Kenya, and HIV-1 RNA levels quantified at the KEMRI/CDC. 

**Figure 1:**
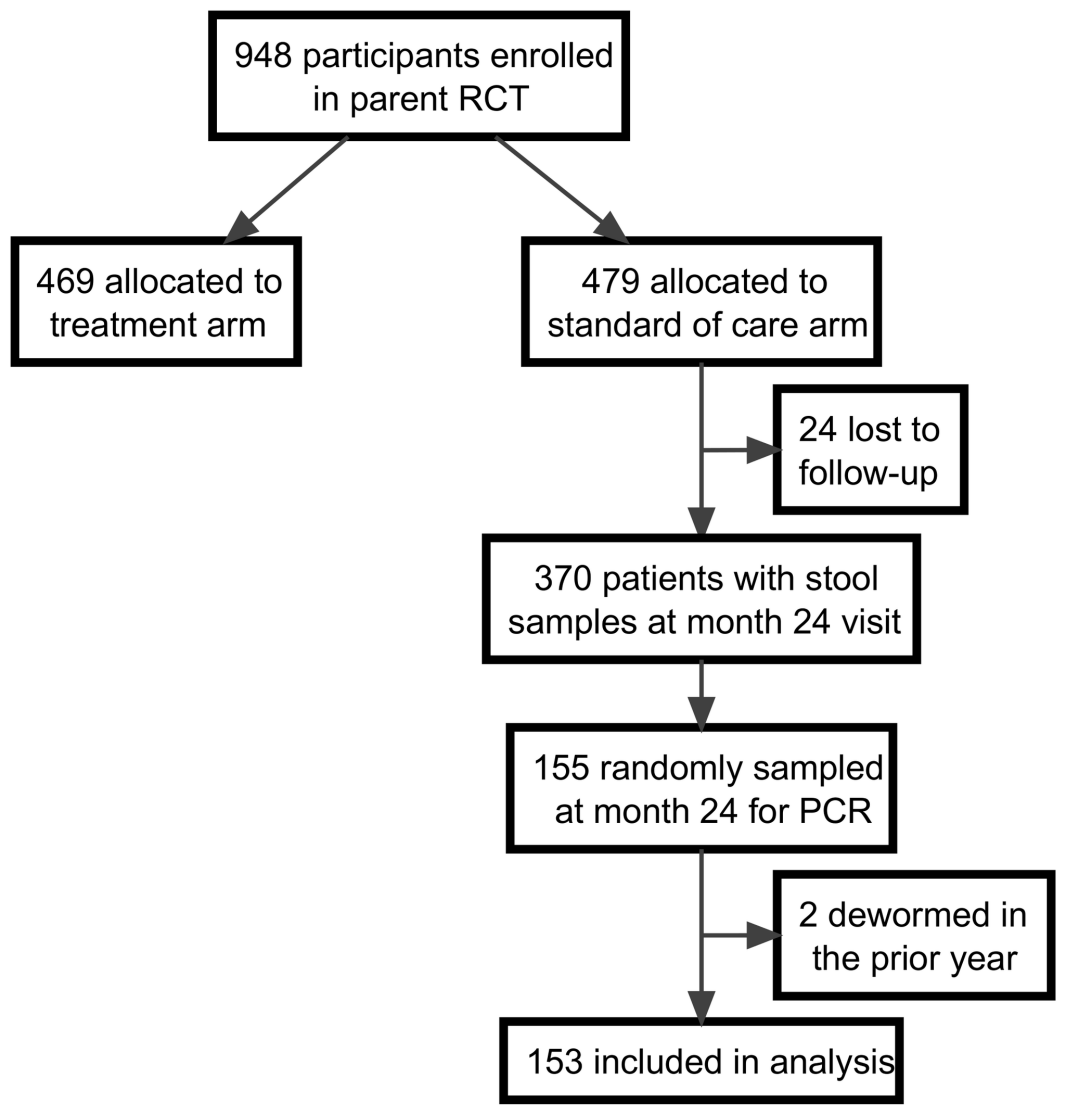
Study flow diagram.

A single stool sample was provided by 740 participants at the final 24 month study visit and was evaluated by technicians with training and certification in the differentiation and quantification of stool helminth species using a combination of wet preparation, Kato-Katz technique (including quantitative assessment of egg counts), and formol-ether concentration within 20 minutes of preparation. Slides were prepared from each sample and were evaluated using each method. For this study, we randomly selected (using a computer-generated algorithm) a subset of 155 individuals with 24 month stool samples from the standard of care arm in the original trial (Figure 1) to receive additional PCR analysis for helminth infection. One gram of unpreserved stool was gathered from each sample within 24 hours of production, sieved, labeled, and stored at -20°C or -80°C in either Greiner 146361 tube or cryo-tube (Corning430659). Stool samples underwent real-time multiplex PCR assessment at the University of Leiden (Leiden, Netherlands) to detect hookworm (*A. duodenale, N. americanus*)*, A. lumbricoides*, *S.* stercoralis*, Schistosoma*-genus[13,32,33] using procedures that have been detailed previously[32,33]. Phocin Herpes Virus 1 (PhHV-1) -specific primers and probe[34] were used as positive controls. 

### Data analysis

Data analysis was performed using STATA version 12.1 IC (College Station, Texas, USA). 

### Diagnostic performance

We calculated sensitivity using PCR and microscopy results; however patients who had taken anthelmintic agents during the past year were excluded (n=2). As there is no true gold standard for helminth diagnosis, individuals identified as positive by PCR or any microscopy method were considered as “true” positives. Sensitivity was calculated for PCR, pooled microscopy, and each microscopy method. Inter-method agreement between microscopy methods and PCR was measured using Cohen’s Kappa statistics. 

### Helminth infection intensity

Fifty PCR amplification cycles were run per sample, and the output was expressed as the cycle threshold (Ct); meaning the amplification cycle in which the level of fluorescent signal exceeds background fluorescence. The Ct reflects species-specific DNA load in the stool samples. Infections were grouped based on DNA load into intensity categories[35] of low (Ct >35), moderate (Ct 30-35), and high (Ct ≤30). Individuals with mixed infections were categorized based on the highest intensity infection. 

### Risk factor and outcome analysis

Baseline risk factors and month 24 clinical outcomes were compared in those with any helminth infection at 24 months versus those without in two series of analyses; first using microscopy detection as the basis for classifying individuals as helminth-infected and second using PCR detection as the basis for classifying individuals as helminth infected. The species-specific microscopy results were combined into a pooled variable in order to enhance helminth detection in the absence of multiple samples[7,10]. Relative risk regression was used to identify univariate risk factors for helminth infection at 24 months, and focused on variables gathered at baseline: CD4 count, HIV plasma viral load, age, number of children in household, education, housing materials, water source, and rural setting. Relative risk regression was used to identify associations between helminth infection at 24 months and dichotomous clinical outcomes gathered at 24 months: diarrhea in the prior 3 months, anemia, and eosinophilia. The coefficients from relative risk regression should be considered prevalence ratios (PR), as we do not know the baseline infection status of patients and the clinical measure analysis was cross-sectional. In this paper we consider the inference from relative risks as a description of associations between variables; therefore the term “risk factor” should be interpreted as a positive association, without implying prediction. Linear regression was used to examine associations between month 24 infection and continuous clinical/laboratory outcomes measured at that visit; including CD4 count, HIV plasma viral load, hemoglobin, hematocrit, eosinophil count, and BMI. Robust standard errors were specified, as values were unlikely to be normally distributed. Anemia status was determined based on standard clinical cutoffs[36]: males were considered anemic if hematocrit <41.0 percent and/or hemoglobin <13.5 g/dl, and females if hematocrit <36.0 percent and/or hemoglobin <12 g/dl[36]. Patients were categorized as having eosinophilia if their eosinophil count was >500 cells/µl[37]. In cases where multicollinearity was a concern, a multivariate regression was run, which included exposure and suspected co-linear variable, and the standard error of the exposure of interest was compared to that of the univariate analysis. Standard errors differing by 10 percent or more were considered suggestive of multicollinearity.

### Immune status and diagnostic accuracy

We used relative risk regression to evaluate the association between month 24 CD4 count >350 cells/mm^3^ and helminth detection by PCR and microscopy. 

### Helminth intensity and outcomes

We investigated the effect of PCR-based infection intensity category on select clinical outcomes. Linear regression was used to investigate eosinophil count, hematocrit percentage, and hemoglobin, and relative risk regression was used for the binary outcomes of anemia and eosinophilia. Additional species-specific intensity analyses were run for schistosomiasis and hookworm infections. Individuals infected with other species were dropped from such analyses. As schistosomiasis-hookworm co-infection was common, species-specific analyses included the second infection intensity (as a continuous Ct value) as a covariate.

## Results

### Patient characteristics

Baseline demographic, socioeconomic, and clinical data from the 153 individuals were summarized and are provided in Table 1. Most participants were women (81.5%) and the median age was 35 years (IQR: 27-41). Over one-third of the cohort had less than a primary education (38.6%) and many subjects (42.8%) reported monthly household incomes of less than 2,000 Kenyan shillings ($23.50). The majority of households utilized a communal water source (55.6%) and most reported using latrines (88.2%) rather than flush toilets (5.9%) or the bush (5.9%). At the time of stool collection (24 month visit of the RCT), the median CD4 count was 365.2 cells/mm^3^. 

**Table 1:** Characteristics of the study participants at baseline (unless otherwise noted) and helminth prevalence at 24 months (N=153).

		**n (%) or median (IQR)**
Sex	Female	124 (81.5)
	Age	35 (27-41)
	Married	86 (56.2)
Education	Less than primary	59 (38.6)
	Primary school	38 (25.5)
	Secondary or higher	55 (35.9)
Occupation	None	35 (22.9)
	Farmer	34 (22.2)
	Other	84 (54.9)
Monthly income (Ksh)	<2,000	65 (42.8)
	2,000-4,999	42 (27.6)
	>5,000	45 (29.6)
	Number of household residents	4 (3-6)
	Number of household residents ages 5-14	1 (0-2)
Water source	Piped water in the house	17 (11.1)
	Communal water source	85 (55.6)
	Environmental water source	17 (11.1)
	Outside water source for household only	34 (22.2)
Sanitation	Bush toilet	9 (5.9)
	Flush toilet	9 (5.9)
	Pit latrine	135 (88.2)
Immune status	Month 0 CD4 T-cell Count (cells/mm^3^)	409.9 (321.5-538.7)
	Month 24 CD4 T-cell Count (cells/mm^3^)	365.2 (267.8-489.5)
Helminth prevalence	Microscopy	20 (13.1)
	PCR	55 (36.0)

### Diagnostic performance

The prevalence of helminth infection in the study was 13.1% as detected using microscopy methods and 36.0% by PCR. Table 2 summarizes the species-specific prevalence and performance of pooled microscopy and PCR. PCR was more sensitive (98.2%) than microscopy (35.7%) (p<0.001). Comparing PCR and pooled microscopy, Cohen’s Kappa statistic was 0.39. All hookworm infections identified by PCR were positive for *N. americanus* and negative for *A. duodenale*.

**Table 2:** Comparison of PCR with pooled microscopy and PCR for species diagnoses (N=153).

	PCR performance^1^		Pooled microscopy performance^1^		
**Helminth Species**	**Positive n (%)**	**Sensitivity$\frac{PCR+}{MIC\;and/or\;PCR+}$**	**Positive n (%)**	**Sensitivity$\frac{MIC+}{MIC\;and/or\;PCR+}$**	**Kappa** ^2^
Any species^3^	55 (36.0)	98.2	20 (13.1)	35.7	0.3896
*A. lumbricoides*	6 (3.9)	85.7	5 (3.3)	71.4	0.7172
Hookworm	31 (20.3)	96.9	10 (6.5)	31.3	0.3775
*S. stercoralis*	5 (3.3)	83.3	1 (0.7)	16.7	-0.0110
*Schistosoma spp.*	20 (13.1)	95.2	6 (3.9)	28.6	0.3451

^1^ Specificity was 100% because each diagnostic method was contained within the “gold-standard”.

^2^ Kappa calculated in comparison to PCR detection *S. stercoralis* (1), counted singly

^3^ 7 individuals co-infected with hookworm and schistosomiasis (6) or *S. stercoralis* (1), counted singly

### Helminth infection intensity

Table 3 summarizes the helminth infection intensity classified by PCR. Among the 55 helminth-infected individuals there were a total of 62 infections: 24 of low intensity, 11 of moderate intensity and 27 of high intensity. There were six individuals infected with both hookworm and schistosomiasis, and one individual infected with hookworm and *S. stercoralis*. The Kato-Katz method identified half (10) of the helminth infections identified by microscopy and therefore egg count information has not been presented.

**Table 3:** Intensity of infection based on PCR DNA load (n= 55).

	**Infection intensity n (%)**			
	**Low (Ct≥35)**	**Moderate (30<Ct<35)**	**High (Ct≤30)**	**Total**
*A. lumbricoides*	0 (0)	2 (3)	4 (7)	6
Hookworm	11 (20)	6 (11)	14 (25)	31
*S. stercoralis*	2 (3)	2 (3)	1 (2)	5
Schistosomiasis	11 (20)	1 (2)	8 (15)	20
Any helminth	24 (39)	11 (18)	27 (44)	62

Infection intensity was positively associated with diagnosis by microscopy. Sixty percent of high intensity infections were detected by microscopy, as compared to only 12% of low intensity infections. The risk of being microscopy-positive for an individual of low, moderate, or high intensity infection was 12.0, 22.7, and 61.0 times that of an individual who was considered uninfected by PCR respectively, with a significant trend test (p<0.001).

### Risk factor analysis

Table 4 summarizes risk factors for helminth infection identified by diagnosis with PCR compared to risk factors identified by microscopy. Using microscopy, no significant associations between helminth infection and the examined risk factors were detected in the cohort. However, PCR diagnosis yielded significant associations between helminth infection and farming occupation (PR 1.57, 95% CI: 1.02, 2.40), communal water source (PR 3.80, 95% CI: 1.01, 14.27), and with lack of primary education (PR 1.54, 95% CI: 1.14, 2.33). 

**Table 4:** Comparison of risk factors° for month 24 helminth infection; cases ascertained by PCR vs. microscopy.

		**n**	**Risk of helminth infection by microscopy (20 cases**)	**Risk of helminth infection by PCR (55 cases**)
			**PR (95% CI)**	**PR (95% CI)**
	Age (per 10 year increase)	153	0.63 (0.38, 1.04)	0.83 (0.66, 1.05)
	No primary education	153	1.95 (0.86, 4.42)	1.54^*^ (1.14, 2.33)
	Farming occupation	153	1.88 (0.82, 4.35)	1.57^*^ (1.02, 2.40)
Water source	Communal	85	-----**^*+*^**	3.80^*^(1.01, 14.27)
	Environmental	17	-----**^*+*^**	2.00 (0.42, 9.50)
	Piped in the house	17	1.00	1.00
	Outside for household only	34	-----**^*+*^**	2.75 (0.69, 11.04)
	Bush toilet use	153	-----**^*!*^**	0.92 (0.36, 2.38)
	Baseline CD4 count<median	153	0.99 (0.44, 2.23)	1.27 (0.83, 1.96)
	Household with ≥1 child 5-14 years old	153	1.07 (0.44, 2.61)	1.11 (0.70, 1.78)
	Household with ≥1 child under age 5	153	1.30 (0.57, 2.97)	1.19 (0.78, 1.82)
	Month 24 CD4 count ≤350 cells/mm^3^	153	1.01 (0.60, 1.53)	1.39 (0.91, 2.13)

° Collected at baseline, with the exception of month 24 CD4 count

^*^ Significant (p<0.05)

^+^ Undefined because there were no microscopy-detected infections in the piped water category

^!^ Undefined because there were no microscopy-detected infections among those who use bush toilets

### Outcome analysis

Table 5 summarizes the analysis of potential clinical and laboratory outcomes from helminth infection, comparing associations in analyses using helminth diagnosis based on PCR to those using microscopy. In analyses comparing individuals with microscopy-diagnosed infections versus without microscopy-detected infection, those with infection had significantly lower hematocrit (mean of -3.56%, 95% CI: -5.85, -1.27), lower hemoglobin (mean of -0.77g/dl, 95% CI: -1.47, -0.07), and 48% higher risk of anemia (PR 1.48, 95% CI: 1.17, 1.88) as compared to individuals considered uninfected. These associations were not observed in analyses comparing infected individuals to those without based on PCR. However, individuals with high intensity (Ct≤30) infections detected by PCR had significantly lower hematocrit than those without high intensity infection (mean of -2.37%, 95% CI: -4.34, -0.40). Significant associations between helminth infection and eosinophilia were also detected in analyses using microscopy (PR 2.92, 95% CI: 1.29, 6.60) and PCR (PR 2.42, 95% CI: 1.02, 5.76). The association was strengthened when individuals with high intensity infections were compared to those without such infections (PR 2.96, 95% CI: 1.32, 6.65). 

**Table 5:** Comparison of clinical outcomes at month 24, month 24 helminth infection; cases ascertained by microscopy vs. PCR vs. high intensity PCR (Ct≤30).

	**n**	**Helminth-infected by microscopy (n=20) Regression coefficient (95% CI)**	**Helminth-infected by PCR (n=55) Regression coefficient (95% CI)**	**High intensity helminth-infected (n=25) Regression coefficient (95% CI)**
Hematocrit (%)	144	-3.56^*^ (-5.85, -1.27)	-1.39 (-3.23, 0.45)	-2.37^*^ (-4.34, -0.40)
Hemoglobin (g/dl)	144	-0.77^*^ (-1.47, -0.07)	-0.24 (-0.85, -0.38)	-0.60 (-1.28, 0.08)
Anemia^4^	144	1.48^*^ (1.17, 1.88)	1.22 (0.95, 1.58)	1.26 (0.95, 1.68)
CD4 T-cell Count (cells/mm^3^)^5^	153	3.38 (-97.17, 103.93)	-21.05 (-82.30, 40.20)	-33.40 (-100.24, 33.46)
Log Viral Load^5^	153	0.21 (-0.21, 0.65)	0.15 (-0.15, 0.46)	-0.33 (-0.08, 0.74)
Eosinophil count (cells/µl)	89	630.92 (-500.20, 1762.04)	314.01 (-185.14, 813.16)	605.85 (-328.08, 1539.8)
Eosinophilia^4^	89	2.92^*^ (1.29, 6.60)	2.42^*^ (1.02, 5.76)	2.96^*^ (1.32, 6.65)
Diarrhea in the past 3 months4(Agarwal and others 2009)	95	1.91 (0.49, 7.40)	0.38 (0.09, 1.62)	0.57 (0.08, 4.08)
2-year change in BMI	147	-0.22 (-1.07, -0.62)	-0.20 (-0.96, 0.56)	-0.41 (-1.20, -0.37)

^*^ Significant (p<0.05)

^4^ Relative risk regression for binary outcome

^5^ Adjusted for ART use

### Immune status and diagnostic accuracy

There was no association between lowered month 24 CD4 count (less than 350 cells/mm^3^) and helminth infection detected by PCR or microscopy (Table 4). The average CD4 count was not significantly different between helminth infected and uninfected individuals using either detection method (Table 5).

### Helminth intensity and outcomes

Helminth infection intensity and hematocrit were significantly associated, as individuals with moderate intensity infection had hematocrit values that were 2.36% lower (95% CI: -4.69, -0.03; p= 0.047) than those who were uninfected, with a significant trend test when treated as a grouped-linear term (p= 0.039). Infection intensity was not significantly associated with hemoglobin or risk of anemia. 

While there was no association between infection intensity and eosinophil count, the risk of eosinophilia was associated with infection intensity, as the risk of eosinophilia among individuals with high intensity infection was 3.4 times that in the uninfected group (PR 3.41, 95% CI: 1.39, 8.37). 

### Species-specific intensity and clinical outcomes

Controlling for schistosomiasis intensity, hookworm intensity was associated with decreased hematocrit (2.42% lower among individuals with moderate infection compared with uninfected, 95% CI: 4.73, -0.13; p=0.039) and possibly hemoglobin (-0.04 g/dl, 95%: -0.07, 0.00; p=0.052). When controlling for hookworm infection intensity, schistosomiasis intensity was associated with a higher risk of eosinophilia; the odds of eosinophilia in those with high intensity schistosomiasis were 9.7 times that in those uninfected (OR 9.70, 95% CI: 1.42, 66.21; p=0.020). There was an association between schistosomiasis intensity and eosinophil count; however this association was with moderate intensity schistosomiasis, a category which contained only one individual. Hookworm infection intensity was not found to be associated with raised eosinophil count or risk of eosinophilia.

## Discussion

PCR markedly increased detection of helminth infections when compared with traditional microscopy in a cohort of HIV-infected adults. The increased sensitivity of the PCR assay resulted in greater power to detect several risk factors significantly associated with helminth infection in the cohort, which were not detected in analyses of the same cohort using stool microscopy. However, PCR-detected infections overall were less likely to be significantly associated with clinical outcomes than microscopy when interpreted as dichotomous, as PCR assay detected many low-burden infections with apparent minimal clinical impact. Alternatively, because of the real-time approach, PCR output could be used to categorize different levels of infection intensity. 

The observed sensitivity for PCR assays and microscopy are consistent with those described previously in other studies[11,13,38,39]. We found that helminths detected by PCR were associated with previously described risk factors for helminth infection, including agricultural occupation[22,23], lack of piped water[22], and low educational level[15,19,21,22]. Importantly, we failed to detect these associations when infection status was determined by microscopy. In previous larger cohorts, microscopy detected risk factors similar to those we detected with PCR assays. However, the use of PCR in this study enabled the detection of risk factors with a smaller sample size, suggesting that sensitive diagnostics improve ability to identify risk factors. We had lower power to detect risk factors with microscopy due to the smaller number of infections.

Microscopy-identified infections were associated with anemia and eosinophilia, suggesting that only higher intensity cases were associated with hematologic and immune dysregulation. This finding is supported by the observed relationship between the risk of eosinophilia and intensity of infection, as the risk of eosinophilia in individuals with high intensity helminth infection was 3.4 times that of uninfected individuals. In addition, among those with high intensity schistosomiasis infection, the odds of eosinophilia were 9.7 times that of uninfected individuals. The significantly lower hematocrit percentages and hemoglobin levels observed in subjects with microscopy-diagnosed infections is consistent with previous studies documenting associations between hookworm infection and anemia[2,27]. However, both moderate and high intensity *A. duodenale* infections detected using real time PCR have previously been shown to be associated with severe anemia among pre-school children in Malawi (adjusted odds ratio: 2.49 and 9.04 respectively)[29]. 

The implications of the data suggest that PCR detects infections of such low intensity that they may be undetectable by traditional methods. While this increased sensitivity may have ramifications for helminth elimination and control efforts, there are several considerations which factor into potential scale-up of PCR diagnostics. The cost of PCR is considerably higher than microscopy methods, and PCR requires levels of laboratory infrastructure and training which are not readily available in many settings. These factors pose challenges which limit current PCR diagnostics to research rather than broader clinical application. Future research should compare the incremental costs of PCR to microscopy.

Strengths of this study include the multi-site sample and parallel comparison of helminth detection techniques. It is the first report to compare the risk factors and outcomes of helminth infection using the two different diagnostic methods, and to assess performance of PCR for helminth infection among HIV-positive adults. The PCR methods used included an internal control to determine efficiency of the PCR and detect inhibition of nucleic acid replication in the sample as reported previously[40]. 

While the study was conducted at several sites in Kenya, there are features that affect the potential external generalizability. The study only included HIV infected adults and was conducted in an area of moderate to low baseline helminth prevalence. The helminth infection burdens were bi-modally distributed, in contrast to the more common overdispersed distribution, in which most individuals harbor a small number of parasites and a small proportion carry the bulk of the parasites[41,42]. Infection status was also not gathered at enrollment, limiting the use of baseline laboratory measurements to control for intra-individual variation. Budget limitations in performing the assays restricted the PCR to a subsample of the patients enrolled in the RCT, half of which were enrolled in the treatment arm (but were excluded from these analyses). While pre-study sample size calculations were performed for the RCT aims, post-hoc power calculations were performed for this secondary analysis. This secondary analysis achieved 80% power to detect a 0.75 g/dl difference in hemoglobin between helminth infected and uninfected subjects, and to detect a prevalence ratio of 1.76 for helminth infection for subjects whose baseline CD4 count was less than the median compared to those above the median. 

Another significant limitation of this study was the microscopy method used to detect parasites. The objective of the parent trial was to evaluate the impact of empiric deworming on HIV progression, and stool testing was only performed to confirm that rates of infection were lower in the treatment arm. As a result, in the parent trial all participants provided a single stool sample at the final study visit which was evaluated using three microscopy methods as described above. These samples were read on site by trained lab technicians but were not subject to verification from a second reader. Given the high variability in day-to-day shedding of eggs, standard stool diagnosis requires testing of multiple stool samples with triplicate Kato-Katz being an optimal microscopy method for helminth diagnosis[8,43]. In a previous study, a single Kato-Katz test had a sensitivity of 17.7% in comparison to repeated collection for detection of S. mansoni and hookworm species[8]. The use of a single stool sample for diagnosis may have substantially underestimated the ability of microscopy to detect infection and may have affected the analyses presented here. Because only 50% of the infections identified by microscopy were from the Kato-Katz technique, we could not evaluate the association between infection intensity determined by PCR Ct and traditional infection intensity expressed as eggs per gram of stool. A standard curve has not been created for such an analysis; however the correlation between hookworm egg count and PCR Ct has been estimated by a previous study (ρ = −0.76; *P* < 0.001)[13].

In summary, our findings suggest that while helminth infections of all intensities are underdiagnosed by microscopy methods, the use of the more sensitive PCR diagnostic test may lead to the detection of a higher proportion of low intensity infections which may be less clinically relevant. PCR can accurately diagnose helminth infections, including low burden infections, and may be useful to discern risk factors and transmission epidemiology. However, the increased diagnostic sensitivity provided by PCR may capture large proportion of lower-intensity infections, which may pose less health risk. Further validation of the quantitative PCR output in different endemic settings is therefore needed. Our study suggests that interpretation of studies on risk factors and outcomes of helminth infection should note the influence of the diagnostic method used to detect infection status.

## References

[1] de Silva NR, Brooker S, Hotez PJ, Montresor A, Engels D et al. (2003) Soil-transmitted helminth infections: updating the global picture. Trends Parasitol 19: 547-551. doi:10.1016/j.pt.2003.10.002. PubMed: 14642761. 14642761

[2] Stoltzfus RJ, Dreyfuss ML, Chwaya HM, Albonico M (1997) Hookworm control as a strategy to prevent iron deficiency. Nutr Rev 55: 223-232. PubMed: 9279058. 9279058 10.1111/j.1753-4887.1997.tb01609.x

[3] Bethony J, Brooker S, Albonico M, Geiger SM, Loukas A et al. (2006) Soil-transmitted helminth infections: ascariasis, trichuriasis, and hookworm. Lancet 367: 1521-1532. doi:10.1016/S0140-6736(06)68653-4. PubMed: 16679166. 16679166

[4] Mahalanabis D, Jalan KN, Maitra TK, Agarwal SK (1976) Vitamin A absorption in ascariasis. Am J Clin Nutr 29: 1372-1375. PubMed: 998548. 998548 10.1093/ajcn/29.12.1372

[5] Brooks RM, Latham MC, Crompton DW (1979) The relationship of nutrition and health to worker productivity in Kenya. East Afr Med J 56: 413-421. PubMed: 520258. 520258

[6] Ndamba J, Makaza N, Munjoma M, Gomo E, Kaondera KC (1993) The physical fitness and work performance of agricultural workers infected with Schistosoma mansoni in Zimbabwe. Ann Trop Med Parasitol 87: 553-561. PubMed: 8122916. 8122916 10.1080/00034983.1993.11812810

[7] Brown M, Bukusuba J, Hughes P, Nakiyingi J, Watera C et al. (2003) Screening for intestinal helminth infestation in a semi-urban cohort of HIV-infected people in Uganda: a combination of techniques may enhance diagnostic yield in the absence of multiple stool samples. Trop Doct 33: 72-76. PubMed: 12680536. 12680536 10.1177/004947550303300206

[8] Booth M, Vounatsou P, N'goran EK, Tanner M, Utzinger J (2003) The influence of sampling effort and the performance of the Kato-Katz technique in diagnosing Schistosoma mansoni and hookworm co-infections in rural Côte d'Ivoire. Parasitology 127: 525-531. doi:10.1017/S0031182003004128. PubMed: 14700188. 14700188

[9] Ebrahim A, El-Morshedy H, Omer E, El-Daly S, Barakat R (1997) Evaluation of the Kato-Katz thick smear and formol ether sedimentation techniques for quantitative diagnosis of Schistosoma mansoni infection. Am J Trop Med Hyg 57: 706-708. PubMed: 9430532. 9430532 10.4269/ajtmh.1997.57.706

[10] Knopp S, Mgeni AF, Khamis IS, Steinmann P, Stothard JR et al. (2008) Diagnosis of soil-transmitted helminths in the era of preventive chemotherapy: effect of multiple stool sampling and use of different diagnostic techniques. PLoS Negl Trop. Drosophila Inf Service 2: e331. 10.1371/journal.pntd.0000331 PMC2570799 18982057

[11] ten Hove RJ, Verweij JJ, Vereecken K, Polman K, Dieye L et al. (2008) Multiplex real-time PCR for the detection and quantification of Schistosoma mansoni and S. haematobium infection in stool samples collected in northern Senegal. Trans R Soc Trop Med Hyg 102: 179-185. doi:10.1016/j.trstmh.2007.10.011. PubMed: 18177680. 18177680

[12] Verweij JJ, Blangé RA, Templeton K, Schinkel J, Brienen EAT et al. (2004) Simultaneous Detection of Entamoeba histolytica, Giardia lamblia, and Cryptosporidium parvum in Fecal Samples by Using Multiplex Real-Time PCR. J Clin Microbiol 42: 1220-1223. doi:10.1128/JCM.42.3.1220-1223.2004. PubMed: 15004079. 15004079 PMC356880

[13] Verweij JJ, Brienen EA, Ziem J, Yelifari L, Polderman AM et al. (2007) Simultaneous detection and quantification of Ancylostoma duodenale, Necator americanus, and Oesophagostomum bifurcum in fecal samples using multiplex real-time PCR. Am J Trop Med Hyg 77: 685-690. PubMed: 17978072. 17978072

[14] Downs JA, Mguta C, Kaatano GM, Mitchell KB, Bang H et al. (2011) Urogenital schistosomiasis in women of reproductive age in Tanzania's Lake Victoria region. Am J Trop Med Hyg 84: 364-369. doi:10.4269/ajtmh.2011.10-0585. PubMed: 21363971. 21363971 PMC3042809

[15] Khalid A, Abdelgadir MA, Ashmaig A, Ibrahim AM, Ahmed AA et al. (2012) Schistosoma mansoni infection among prenatal attendees at a secondary-care hospital in central Sudan. Int J Gynaecol Obstet 116: 10-12. doi:10.1016/j.ijgo.2011.08.018. PubMed: 22036060. 22036060

[16] Knopp S, Mohammed KA, Stothard JR, Khamis IS, Rollinson D et al. (2010) Patterns and risk factors of helminthiasis and anemia in a rural and a peri-urban community in Zanzibar, in the context of helminth control programs. PLoS Negl Trop. Drosophila Inf Service 4: e681. 10.1371/journal.pntd.0000681 PMC2867941 20485491

[17] Sarkinfada F, Oyebanji AA, Sadiq IA, Ilyasu Z (2009) Urinary schistosomiasis in the Danjarima community in Kano, Nigeria. J Infect Dev Ctries 3: 452-457. PubMed: 19762959. 19762959 10.3855/jidc.417

[18] van Eijk AM, Hill J, Alegana VA, Kirui V, Gething PW et al. (2011) Coverage of malaria protection in pregnant women in sub-Saharan Africa: a synthesis and analysis of national survey data. Lancet Infect Dis 11: 190-207. doi:10.1016/S1473-3099(10)70295-4. PubMed: 21273130. 21273130 PMC3119932

[19] Woodburn PW, Muhangi L, Hillier S, Ndibazza J, Namujju PB et al. (2009) Risk factors for helminth, malaria, and HIV infection in pregnancy in Entebbe, Uganda. PLoS Negl Trop. Drosophila Inf Service 3: e473. 10.1371/journal.pntd.0000473 PMC2696595 19564904

[20] Ndassa A, Mimpfoundi R, Gake B, Paul Martin MV, Poste B (2007) Risk factors for human schistosomiasis in the Upper Benue valley, in northern Cameroon. Ann Trop Med Parasitol 101: 469-477. PubMed: 17716429. 17716429 10.1179/136485907X193752

[21] Olsen A, Samuelsen H, Onyango-Ouma W (2001) A study of risk factors for intestinal helminth infections using epidemiological and anthropological approaches. J Biosoc Sci 33: 569-584. doi:10.1017/S0021932001005697. PubMed: 11683225. 11683225

[22] Walson JL, Stewart BT, Sangaré L, Mbogo LW, Otieno PA et al. (2010) Prevalence and correlates of helminth co-infection in Kenyan HIV-1 infected adults. PLoS Negl Trop. Drosophila Inf Service 4: e644. 10.1371/journal.pntd.0000644 PMC2846937 20361031

[23] Humphries D, Mosites E, Otchere J, Twum WA, Woo L et al. (2011) Epidemiology of hookworm infection in Kintampo North Municipality, Ghana: patterns of malaria coinfection, anemia, and albendazole treatment failure. Am J Trop Med Hyg 84: 792-800. doi:10.4269/ajtmh.2011.11-0003. PubMed: 21540391. 21540391 PMC3083749

[24] Belyhun Y, Medhin G, Amberbir A, Erko B, Hanlon C et al. (2010) Prevalence and risk factors for soil-transmitted helminth infection in mothers and their infants in Butajira, Ethiopia: a population based study. BMC Public Health 10: 21. doi:10.1186/1471-2458-10-21. PubMed: 20085635. 20085635 PMC2835680

[25] Modjarrad K, Zulu I, Redden DT, Njobvu L, Freedman DO et al. (2005) Prevalence and predictors of intestinal helminth infections among human immunodeficiency virus type 1-infected adults in an urban African setting. Am J Trop Med Hyg 73: 777-782. PubMed: 16222025. 16222025 PMC2749260

[26] Stoltzfus RJ (2001) Iron-deficiency anemia: reexamining the nature and magnitude of the public health problem. Summary: implications for research and programs. J Nutr 131: 697S-701S; discussion: 11160600 10.1093/jn/131.2.697S

[27] Smith JL, Brooker S (2010) Impact of hookworm infection and deworming on anaemia in non-pregnant populations: a systematic review. Trop Med Int Health 15: 776-795. doi:10.1111/j.1365-3156.2010.02542.x. PubMed: 20500563. 20500563 PMC2916221

[28] Brooker S, Hotez PJ, Bundy DA (2008) Hookworm-related anaemia among pregnant women: a systematic review. PLoS Negl Trop. Drosophila Inf Service 2: e291. 10.1371/journal.pntd.0000291 PMC2553481 18820740

[29] Jonker FA, Calis JC, Phiri K, Brienen EA, Khoffi H et al. (2012) Real-time PCR demonstrates Ancylostoma duodenale is a key factor in the etiology of severe anemia and iron deficiency in Malawian pre-school children. PLoS Negl Trop. Drosophila Inf Service 6: e1555. 10.1371/journal.pntd.0001555 PMC3295794 22514750

[30] Karanja DM, Colley DG, Nahlen BL, Ouma JH, Secor WE (1997) Studies on schistosomiasis in western Kenya: I. Evidence for immune-facilitated excretion of schistosome eggs from patients with Schistosoma mansoni and human immunodeficiency virus coinfections. Am J Trop Med Hyg 56: 515-521. PubMed: 9180601. 9180601 10.4269/ajtmh.1997.56.515

[31] Walson J, Singa B, Sangaré L, Naulikha J, Piper B et al. (2012) Empiric deworming to delay HIV disease progression in adults with HIV who are ineligible for initiation of antiretroviral treatment (the HEAT study): a multi-site, randomised trial. Lancet Infect Dis 12: 925-932. doi:10.1016/S1473-3099(12)70207-4. PubMed: 22971323. 22971323

[32] Obeng BB, Aryeetey YA, de Dood CJ, Amoah AS, Larbi IA et al. (2008) Application of a circulating-cathodic-antigen (CCA) strip test and real-time PCR, in comparison with microscopy, for the detection of Schistosoma haematobium in urine samples from Ghana. Ann Trop Med Parasitol 102: 625-633. doi:10.1179/136485908X337490. PubMed: 18817603. 18817603

[33] Wiria AE, Prasetyani MA, Hamid F, Wammes LJ, Lell B et al. (2010) Does treatment of intestinal helminth infections influence malaria? Background and methodology of a longitudinal study of clinical, parasitological and immunological parameters in Nangapanda, Flores, Indonesia (ImmunoSPIN Study). BMC Infect Dis 10: 77. doi:10.1186/1471-2334-10-77. PubMed: 20338054. 20338054 PMC2859773

[34] Niesters HG (2002) Clinical virology in real time. J Clin Virol 25 Suppl 3: S3-12. doi:10.1016/S1386-6532(02)00026-4. PubMed: 12467772. 12467772 PMC7128152

[35] Wiria AE, Hamid F, Wammes LJ, Kaisar MM, May L et al. (2013) The Effect of Three-Monthly Albendazole Treatment on Malarial Parasitemia and Allergy: A Household-Based Cluster-Randomized, Double-Blind, Placebo-Controlled Trial. PLOS ONE 8: e57899. doi:10.1371/journal.pone.0057899. PubMed: 23526959. 23526959 PMC3602425

[36] Schrier S (2012) Approach to the adult patient with anemia UpToDate.

[37] Tefferi A (2005) Blood eosinophilia: a new paradigm in disease classification, diagnosis, and treatment. Mayo Clin Proc 80: 75-83. doi:10.1016/S0025-6196(11)62962-5. PubMed: 15667033. 15667033

[38] Pontes LA, Dias-Neto E, Rabello A (2002) Detection by polymerase chain reaction of Schistosoma mansoni DNA in human serum and feces. Am J Trop Med Hyg 66: 157-162. PubMed: 12135287. 12135287 10.4269/ajtmh.2002.66.157

[39] Pontes LA, Oliveira MC, Katz N, Dias-Neto E, Rabello A (2003) Comparison of a polymerase chain reaction and the Kato-Katz technique for diagnosing infection with Schistosoma mansoni. Am J Trop Med Hyg 68: 652-656. PubMed: 12887022. 12887022

[40] Monteiro L, Bonnemaison D, Vekris A, Petry KG, Bonnet J et al. (1997) Complex polysaccharides as PCR inhibitors in feces: Helicobacter pylori model. J Clin Microbiol 35: 995-998. PubMed: 9157172. 9157172 10.1128/jcm.35.4.995-998.1997 PMC229720

[41] Guyatt HL, Bundy DA, Medley GF, Grenfell BT (1990) The relationship between the frequency distribution of Ascaris lumbricoides and the prevalence and intensity of infection in human communities. Parasitology 101 1: 139-143. doi:10.1017/S0031182000079841. PubMed: 2235069. 2235069

[42] Croll NA, Ghadirian E (1981) Wormy persons: contributions to the nature and patterns of overdispersion with Ascaris lumbricoides, Ancylosotma duodenale, Necator americanus and Trichuris trichiura. Trop Geogr Med 33: 241-248. PubMed: 7314236. 7314236

[43] Sturrock RF (1998) Guidelines for the evaluation of soil-transmitted helminthiasis and schistosomiasis at community level: A guide for managers of control programmes: A. Montresor, D. W. T. Crompton, D. A. P. Bundy, A. Hall & L. Savioli. Geneva: World Health Organization , 1998. iv+46pp. Transactions of the Royal Society of Tropical Medicine and Hygiene 92: 470-471

